# Stability and Antiglycoxidant Potential of Bilberry Anthocyanins in Simulated Gastrointestinal Tract Model

**DOI:** 10.3390/foods9111695

**Published:** 2020-11-19

**Authors:** Didier Fraisse, Alexis Bred, Catherine Felgines, François Senejoux

**Affiliations:** Unité de Nutrition Humaine, Université Clermont-Auvergne, INRA, UNH, CRNH Auvergne, F-63000 Clermont-Ferrand, France; didier.fraisse@uca.fr (D.F.); alexis.bred@uca.fr (A.B.); catherine.felgines@uca.fr (C.F.)

**Keywords:** bilberry, anthocyanin, digestion, antioxidant, glycation, bioaccessibility

## Abstract

Anthocyanins from *Vaccinium myrtillus* fruits have been reported in vitro to exert potent radical scavenging and antiglycation activities. However, the physiological relevance of such properties remains unclear given the potential susceptibility of anthocyanin derivatives to digestive conditions. A simulated gastrointestinal tract model was thus implemented to assess the impact of gastric and intestinal phases on the chemical integrity of bilberry anthocyanins and their antiglycoxidant effects. Results demonstrated that the investigated activities as well as total and individual anthocyanin contents were marginally affected by gastric conditions. By contrast, with recoveries ranging from 16.1 to 41.2%, bilberry anthocyanins were shown to be highly sensitive to the intestinal phase. Of major interest, a much better preservation was observed for radical scavenging and antiglycation activities as attested by recovery rates ranging from 79.1 to 86.7%. Consistently with previous observations, the present study confirms the moderate bioaccessibility of anthocyanin constituents. It does however provide valuable information supporting the persistence of substantial radical scavenging and antiglycation activities at each step of the digestion process. Taken together, these data indicate that digestive conditions might not abolish the potential positive effects of bilberry consumption on both oxidative and carbonyl stresses.

## 1. Introduction

*Vaccinium myrtillus* L. (Ericaceae) is a deciduous low growing shrub that produces dark purple edible berries. Known also as bilberry, its fruits are regarded as one of the richest natural sources of anthocyanins, with contents amounting to approximately 0.5% in fresh material [1]. With more than 700 constituents reported so far [2], anthocyanins constitute an important group of phenolic compounds belonging to the family of flavonoids. These water-soluble pigments are accountable for the red, pink, blue, and purple colors of numerous flowers and fruits. Most of them occur as glycosides in nature and are derived from six principal aglycones moieties that include cyanidin, delphinidin, malvidin, pelargonidin, peonidin, and petunidin [3]. Of interest, bilberry has been reported to contain a substantial diversity of anthocyanin structures, and all the major anthocyanidins are represented with the sole exception of pelargonidin [4].

It is worth mentioning that bilberry extracts as well as anthocyanin constituents have been shown in vitro to exert diverse biological activities including potent radical scavenging and antiglycative effects [5,6]. Of interest, these two reported properties might account for the health benefits of bilberry supplementation on numerous oxidative and carbonyl stress-related diseases [7,8]. Preventive effects on several chronic pathologies, such as cardiovascular diseases and diabetes, have been indeed pointed out by multiple clinical investigations [8,9]. However, it should be noted that anthocyanin derivatives have been reported to undergo extensive metabolism during digestion process [10,11]. This phenomenon might be explained, at least in part, by the impact of intestinal pH on their structural integrity [11]. Indeed, the limited stability of these constituents in neutral or slightly basic aqueous media have been clearly demonstrated [12,13]. Of interest, several investigations aimed at characterizing some of the major decomposition products. It has been reported that a wide range of phenolic acid derivatives can be generated, including protocatechuic acid, gallic acid, vanillic acid, syringic acid, and 4-hydroxycinnamic acid [13]. Besides, the formation of 2,4,6-trihydroxybenzaldehyde has also been documented [14]. Given this context, it remains unclear to what extent the reported in vitro activities of bilberry anthocyanins are physiologically relevant. Further evidence is actually required to validate the persistence of their antioxidant and antiglycative effects after digestion-induced modifications.

Of interest, several in vitro digestion methods have been recently developed to mimic the conditions of gastrointestinal tract [15]. By using digestive enzymes at physiological concentrations and by controlling factors such as pH value, salt content and digestion time, these approaches are able to simulate the most important steps of the digestion process. Such in vitro models have been notably employed to assess the digestibility and bioaccessibility of a wide range of pharmaceutical drugs, toxins, and macronutrients [15]. In addition, they have also been successfully implemented to determine the digestive fate of numerous plant extracts and food bioactives [16,17]. However, to our knowledge, data are lacking regarding bilberry extract and its anthocyanin components. The present study aimed thus at employing a standardized in vitro model to evaluate the impact of digestive conditions on an anthocyanin-rich extract from *V. myrtillus* fruits (AEVM). Different sets of experiments were instigated to assess the influence of gastric and intestinal phases on the chemical composition as well as the bioactivity of the studied extract. Colorimetric estimations of its Total Phenolic (TPC) and Total Anthocyanin Contents (TAC) were first performed at each step of the simulated digestion process. Besides, the digestive fate of its anthocyanin constituents was also individually assessed by means of HPLC-UV analyses. Finally, bovine serum albumin (BSA)/D-ribose evaluation as well as 2,2-diphenyl-1-picrylhydrazyl (DPPH) and 2,2’-azino-bis 3-ethylbenzothiazoline-6-sulphonic acid (ABTS) radical scavenging assays were employed to validate the persistence of bilberry antiglycoxidant activity in simulated gastrointestinal conditions.

## 2. Materials and Methods

### 2.1. Plant Material and Reagents

Bilberry anthocyanin-rich extract, Anthocyan^®^, was provided by Ferlux SA (Cournon d’Auvergne, France). Methanol and acetonitrile (MeCN) were of chromatographic grade and were purchased from Carlo Erba Reagents SAS (Val-de-Reuil, France). All aqueous solutions were prepared with pure water produced by Milli-Q water (18.2 MΩ) system (Merck, Darmstadt, Germany). Phosphoric acid (85%), hydrochloric acid (HCl, 37% *w/w*) and sodium hydroxide (NaOH) were bought from VWR Prolabo (Fontenay-sous-Bois, France). Cyanidin 3-*O*-glucoside was purchased from Extrasynthese (Genay, France). Pancreatin from porcine pancreas (8 × USP specification), pepsin from porcine gastric mucosa (lyophilized powder, 3200-4500 units/mg protein), BSA, DPPH, ABTS, D-ribose, Folin-Ciocalteu’s reagent, gallic acid, 6-hydroxy-2,5,7,8-tetramethylchromane-2-carboxylic acid (Trolox), sodium chloride, calcium chloride dihydrate, potassium chloride, magnesium chloride hexahydrate, potassium phosphate monobasic, sodium bicarbonate, and ammonium carbonate were obtained from Sigma–Aldrich Chemical (Saint-Quentin Fallavier, France). DPPH and ABTS radical solutions were respectively prepared every day and every half-day and were kept protected from light at 4 °C.

### 2.2. In Vitro Gastrointestinal Digestion Procedure

**Figure 1:**
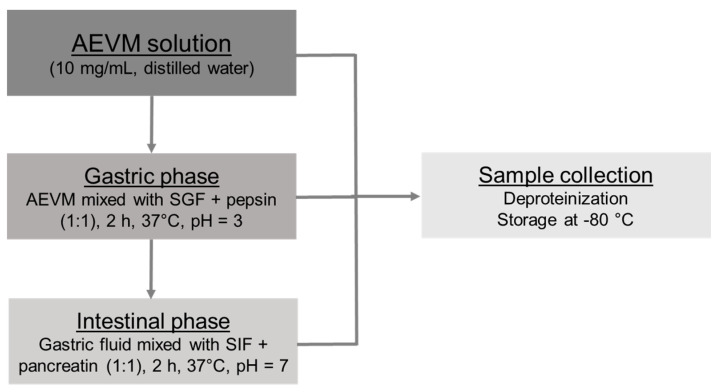
Flow diagram of the simulated in vitro digestion procedure. AEVM: Anthocyanin enriched extract from *Vaccinium myrtillus*, SGF: Simulated Gastric Fluid, SIF: Simulated Intestinal Fluid.

In vitro digestion protocol (Figure 1) was performed according to the standardized method reported by Minekus et al. [15]. Of note, only gastric and intestinal phases were included. Preparation and composition of Simulated Gastric Fluid (SGF) and Simulated Intestinal Fluid (SIF) electrolyte stock solutions strictly followed the procedure of Minekus et al. [15] and identical dilutions were achieved during in vitro digestion experiments. Adaptations regarding sample collection and handling options were, however, operated. All digestion experiments were performed in triplicate (*n* = 3).

#### 2.2.1. Gastric Phase

Regarding gastric step, 5 mL of AEVM solution (10 mg/mL in distilled water) was mixed with 3 mL of SGF electrolyte stock solution and 1 mL of pepsin solution made up in SGF stock solution (20,000 U/mL). A final volume of 10 mL was obtained after addition of calcium chloride (0.075 mM in final gastric mixture), water, and pH adjustment to 3.0 with 1 M HCl. A two hours incubation at 37 °C was performed with constant shaking at 50 rpm in an orbital shaking incubator (NB-205 L, N-Biotek, Bucheon-si, South Korea). The obtained gastric mixture was divided in two equal parts, 5 mL was employed for intestinal digestion, and 5 mL was reserved for chemical and biological evaluations.

#### 2.2.2. Intestinal Phase

For intestinal phase, 5 mL of gastric mixture was mingled with 3 mL of SIF stock solution and 1 mL of pancreatin solution made up in SIF stock solution (1000 U/mL). A final volume of 10 mL was obtained after addition of calcium chloride (0.3 mM in final intestinal mixture), water, and pH adjustment to 7.0 using 0.1 M NaOH. Similarly to gastric phase, a 2 h incubation was performed using a shaking incubator (37 °C, 50 rpm).

#### 2.2.3. Sample Management

Gastric samples were first diluted in distilled water (1/2) to normalize AEVM concentration among different samples. Gastric and intestinal samples were immediately deproteinized by adding 4 volumes of ethanol and were centrifugated at 4300 rpm for 15 min (Centrifuge 5804 R, Eppendorf, Montesson, France). Supernatants were then divided in aliquots of 1 mL, which were stored at −80 °C until further analysis. Undigested sample of AEVM was also prepared (2.5 mg/mL in distilled water) to serve as reference. It was submitted to equivalent deproteinization, centrifugation and conservation than digestive samples.

### 2.3. Spectrometric Evaluations of Total Phenolic Content, Total Anthocyanin Content, Radical Scavenging, and Antiglycation Activities

TPC was determined according a previously reported method with slight modifications [18]. Briefly, reference and digestive retreated samples were diluted five times in distilled water, and 2 mL of the obtained solutions was mingled with 1 mL of undiluted Folin Ciocalteu’s reagent. The volume was finally adjusted to 25 mL with a sodium carbonate solution (150 g/L). After an incubation of 30 min at room temperature, the absorbance was recorded at 740 nm using a Jasco V-630 spectrophotometer (Lisses, France). A standard curve of gallic acid with concentrations ranging from 5 to 100 μg/mL was constructed (*R*^2^ = 0.9985, y = 4.418 x + 0.020) and TPC was expressed as mg of gallic acid equivalents (mg GAE) per g of dry extract. TAC was evaluated using colorimetric method following European Pharmacopoeia procedure [19], with slight adaptations. A 25 x dilution of reference and digestive samples was done with methanolic HCl (0.1%), and absorbance was evaluated at 528 nm with a Jasco V-630 spectrophotometer. A standard curve of cyanidin 3-*O*-glucoside (5–100 μg/mL) was realized (*R*^2^ = 0.9921, y = 65.116 x + 0.011). The amount of total anthocyanins was expressed as milligram of cyanidin 3-*O*-glucoside equivalent per gram of dry extract.

DPPH radical scavenging capacity was evaluated according to a previously published protocol [20], with minor adaptations. Briefly, reference and digestive retreated samples were diluted 10 times in distilled water. Then, 20 µL of the obtained diluted solutions was mixed to 2.5 mL of fresh DPPH solution (25 μg/mL in methanol). After 30 min of incubation at room temperature, absorbance was measured at 515 nm using a UV–vis Jasco V-630 spectrophotometer. A standard curve of Trolox (100–3000 μmol/L) was plotted (*R*^2^ = 0.9972, y = 1081.4 x + 1.750), and DPPH radical scavenging activities were expressed in micromoles of Trolox equivalent (μmol TE) per gram of dry extract.

ABTS radical scavenging activity was estimated following a previous published protocol [21], with slight modifications. Retreated samples (reference and digestive solutions) were diluted 10 times with distilled water. Then, 20 µL of the obtained solutions was combined with 250 µL of ABTS^+•^ solution, freshly prepared following the procedure of Re et al. [22]. After 10 min of incubation, the decrease in absorbance was measured at 734 nm with a microplate reader (TECAN infinite F200 PRO microplate reader, Lyon, France). Similarly with DPPH evaluation, a standard curve of Trolox (100–2400 μmol/L) was constructed (*R*^2^ = 0.992, y = 11760 x + 0.5495) and ABTS radical scavenging capacities were indicated in μmol TE/g of dry extract.

Inhibition of Advanced Glycation End-products (AGEs) formation was determined using BSA/D-ribose assay as previously reported [18,23]. AGEs fluorescence was assessed using a microplate reader (TECAN infinite F200 PRO) with 370 and 440 nm as the excitation and emission wavelengths. Experiments were performed on at least six different dilutions of undigested and digested retreated samples. Results were expressed as IC_50_ in mg of dry extract/L. Recovery index of antiglycation activity was calculated by comparing 1/IC_50_ values of gastric and intestinal solutions to that of undigested matrix.

### 2.4. HPLC Analysis of AEVM

HPLC analyses were done using a LaChrom Elite system (VWR-Hitachi, Radnor, Pennsylvania, USA) equipped with two L7100 pumps, a L7200 autosampler, a L2450 diode array detector (DAD), and EZ Chrom Elite software. Undigested and digested retreated samples were diluted five times and then chromatographed using a reversed phase Purospher^®^ Star C_8_ endcapped column (125 × 4 mm, 5 μm particle size). A gradient elution was employed with a mobile phase composed of water containing 1% phosphoric acid (A) and MeCN (B). The gradient was set as follows: 0–5 min, 5% B; 5–30 min, 5–7% B; 30–45 min, 7–12% B; 45–50 min, 12–40% B. A flow rate of 1 mL/min, an injection volume of 50 μL, and a monitoring wavelength of 530 nm were selected.

### 2.5. Statistical Analyses

The statistical significance of difference was analyzed by one-way ANOVA followed by Fisher’s Least Significant Difference (LSD) test and *p* values of 0.05 or less (*p* ≤ 0.05) were considered statistically significant. All data are indicated as mean ± standard error of mean (SEM). All spectrometric and fluorimetric analyses were done in triplicate (*n* = 3).

## 3. Results and Discussion

### 3.1. Recovery Index of Total Anthocyanin and Total Phenolic Contents

Bearing in mind that anthocyanins are regarded as important contributors to the health benefit of bilberry consumption [7], the present study focused on the digestive fate of that chemical group. As indicated in Table 1, spectrometric analyses of TPC and TAC of AEVM undigested solution revealed respective concentrations of 434.8 ± 1.9 mg GAE and 321.8 ± 2.0 mg cyanidin 3-*O*-glucoside equivalent/g of dry extract, thus highlighting a substantial ratio of anthocyanin constituents. Besides, these two contents were evaluated after gastric and intestinal simulated digestions.

**Figure 2:**
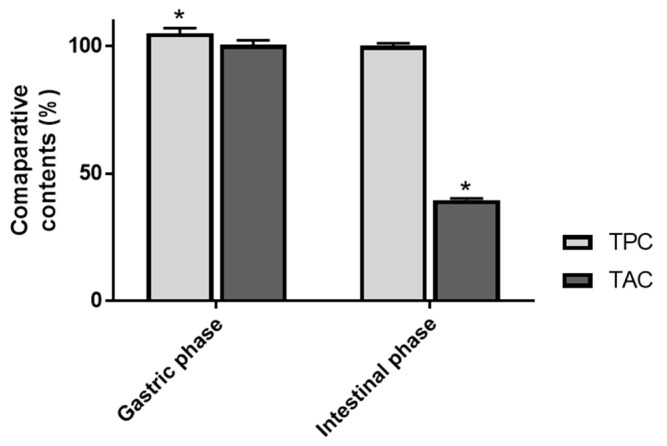
Impact of in vitro gastrointestinal digestion on Total Phenolic Content (TPC) and Total Anthocyanin Content (TAC) of bilberry extract. Data are presented as means ± SEM (*n* = 3). All results are expressed as percentages, with control (i.e., undigested matrix) normalized as 100%. * *p* < 0.05 vs. control.

Interestingly, gastric phase induced negligible modifications in both TPC and TAC. Indeed, with a quantitative recovery of 100.5 ± 1.8%, anthocyanin content of bilberry appeared not to be significantly affected by gastric simulated conditions (Figure 2). It has to be noted that this result is consistent with previous investigations of the impact of in vitro digestion on other anthocyanin rich extracts. For instance, anthocyanin global contents of red wine [24], red cabbage [25], or cornelian cherry fruit [26] were also determined to be well preserved after simulated gastric step. Such observations are in line with the established stability of anthocyanin derivatives in acidic conditions. Additionally, it tends to indicate that gastric enzymes do not exert noticeable metabolizing activity on that class of constituents. Regarding TPC, a slight but significant increase was assessed following gastric step (105.0 ± 2.0%). The creation of additional phenolic derivatives during simulated digestion process appears highly unlikely. However, this marginal augmentation might be related to structural modulations affecting some minor phenolic components other than anthocyanin derivatives. It can very well be envisaged that such modifications result in a perceptible augmentation of their reducing capacity and their reactivity toward Folin-Ciocalteu reagent. Of interest, several other in vitro digestion evaluations of phenolic containing plant extracts have also reported analogous results. For instance, in vitro investigations of the digestive fate of *Mimosa scabrella*, *Lippia graveolens*, and *Hedeoma patens* have also pointed out an increase in their TPC following gastric step [16,27].

By contrast, intestinal phase induced a significant diminution of anthocyanin concentration (*p* < 0.05). Indeed, with an estimated reduction of 60.4 ± 0.6%, bilberry TAC was shown to be deeply affected by this second digestive phase. Of note, this value is in the same order of magnitude as those reported in previous in vitro digestion studies. Decrease rates ranging from 55 to 90% have effectively been highlighted for several other anthocyanin containing extracts [25,26,28]. This phenomenon might be explained, at least partly, by the impact of intestinal pH on the structural integrity of anthocyanins. Indeed, the limited stability of these constituents in neutral or slightly basic aqueous media have been highlighted by several evaluations [12,13]. Contrarily to anthocyanin content, it is interesting to note that TPC was not significantly modified by intestinal simulated conditions, as attested by its recovery value of 100.9 ± 0.9%. It clearly indicates that degradation products of anthocyanins are still corresponding to phenolic entities. Besides, it suggests that parent anthocyanins and their decomposition components tend to similarly react with Folin reagent.

### 3.2. Individual Stability of Anthocyanin Constituents during Simulated In Vitro Gastrointestinal Digestion

**Figure 3:**
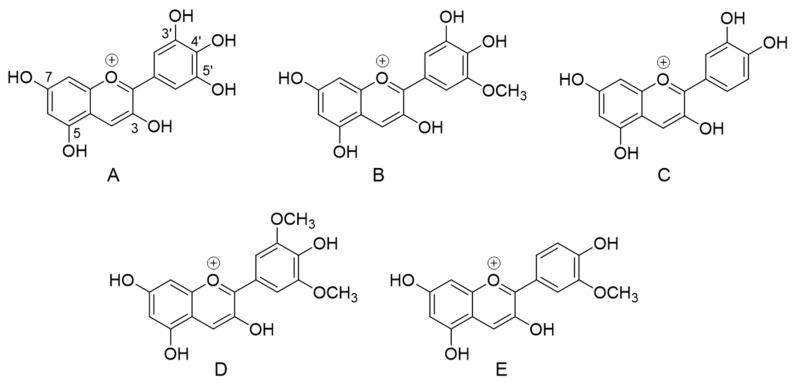
Chemical structures of the anthocyanidin aglycones occurring in *Vaccinium myrtillus* fruit. (**A**) delphinidin, (**B**) petunidin, (**C**) cyanidin, (**D**) malvidin, (**E**) peonidin.

**Figure 4:**
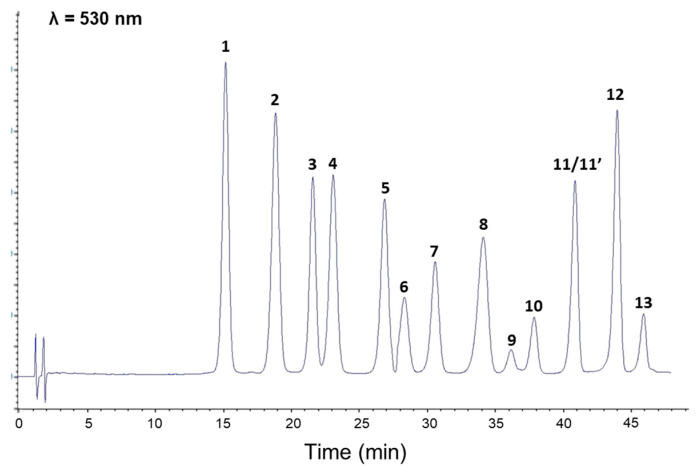
HPLC profile of *Vaccinium myrtillus* fruit extract. 1: delphinidin 3-*O*-galactoside, 2: delphinidin 3-*O*-glucoside, 3: cyanidin 3-*O*-galactoside, 4: delphinidin 3-*O*-arabinoside, 5: cyanidin 3-*O*-glucoside, 6: petunidin 3-*O*-galactoside, 7: cyanidin 3-*O*-arabinoside, 8: petunidin 3-*O*-glucoside, 9: peonidin 3-*O*-galactoside, 10: petunidin 3-*O*-arabinoside, 11: peonidin 3-*O*-glucoside, 11′: malvidin 3-*O*-galactoside, 12: malvidin 3-*O*-glucoside, 13: malvidin 3-*O*-arabinoside.

The aforementioned evaluation of total anthocyanin content has highlighted the substantial impact of intestinal phase on this class of constituents. Nonetheless, it has been previously demonstrated that stability and digestive recovery of anthocyanin derivatives significantly differ depending on their chemical structures [29]. Considering the structural diversity of bilberry anthocyanins (Figure 3), HPLC-DAD experiments were thus implemented to specifically assess the impact of gastrointestinal tract on each component of AEVM. Consistently with previous chemical investigations of this same extract [4,5], 13 main anthocyanin peaks were detected (Figure 4), and five different groups were represented including delphinidin (1, 2, and 4), cyanidin (3, 5, and 7), petunidin (6, 8, and 10), malvidin (11′, 12, and 13), and peonidin glycosides (9 and 11). Of note, a co-elution occurred between peonidin 3-O-glucoside and malvidin 3-O-galactoside (11 and 11′).

Coherently with TAC evaluation, data showed that gastric phase did not markedly affect anthocyanin constituents. As illustrated in Table 2, recovery values higher than 96% were calculated for all components, thus validating the very good stability of bilberry anthocyanins in gastric conditions.

**Figure 5:**
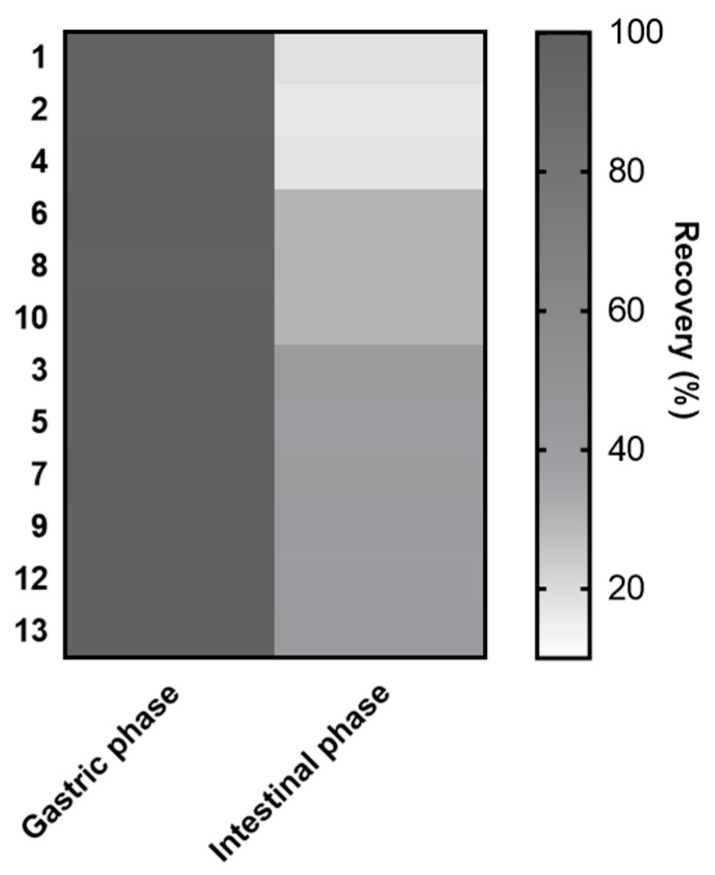
Heat map representation of the recovery index of anthocyanins from *Vaccinium myrtillus* fruit extract clustered according to their aglycone moieties: delphinidin (1, 2, 4), petunidin (6, 8, 10), cyanidin (3, 5, 7), peonidin (9), and malvidin (12, 13).

More importantly, anthocyanin analysis in intestinal simulated media confirmed the striking impact of that digestive phase. Indeed, decrease rates ranging from 58.8 to 83.9% were assessed for all constituents. Of interest, similar reductions were spotted among constituents with analogous aglycones. It thus suggests that the nature of the sugar moiety might not profoundly influence the sensitivity of bilberry anthocyanins to intestinal simulated conditions. By contrast, substantial differences were observed depending on anthocyanidin moieties (Figure 5). Indeed, with respective diminutions of 81.6, 83.9, and 82.6%, delphinidin glycosides (1, 2, 4) were shown to be the most affected constituents of the extract. Besides, petunidin derivatives (6, 8, 10) were also highly impacted by intestinal conditions as attested by their reduction rates of around 70%. Conversely, cyanidin (3, 5, 7) peonidin (9), and malvidin (12, 13) constituents were all determined to possess a slightly superior stability in simulated intestinal conditions, with diminutions ranging from 58.8 to 61.8%. It is worth mentioning that similar tendencies were previously reported by Yang et al. [13]. Indeed, this investigation of the impact of in vitro digestion on five representative anthocyanin glucosides also revealed that delphinidin and petunidin 3-*O*-glucosides were significantly more affected by intestinal phase than malvidin, peonidin, and cyanidin 3-*O*-glucosides.

### 3.3. Impact of Simulated Digestion on Radical Scavenging and Antiglycation Activities of AEVM

Owing to their hydroxyl substituents and their aromatic structure, numerous phenolic constituents have been shown to exert pronounced radical scavenging activities as well as promising inhibitory action on AGEs formation [30,31]. These properties are assumed to play an important role in their preventive effects against several degenerative and chronic pathologies. Of interest, several studies have emphasized the remarkable in vitro antiglycative and antioxidant properties of berry anthocyanins [32,33]. Nevertheless, such evaluations might not be sufficient to fully validate their health benefits since the potential influence of digestive processes is not taken into account. Besides, the above chemical data underline the modest bioaccessibility of anthocyanin constituents as well as the strong impact of intestinal conditions on their structural integrity. Additional investigations are thus required to confirm whether bilberry antiglycoxidant properties do persist after digestion induced alteration of its bioactive components. As indicated in Table 3, DPPH and ABTS radical scavenging evaluations demonstrated that gastric simulated phase did not exert a negative influence on the antioxidant properties of the studied extract.

**Figure 6:**
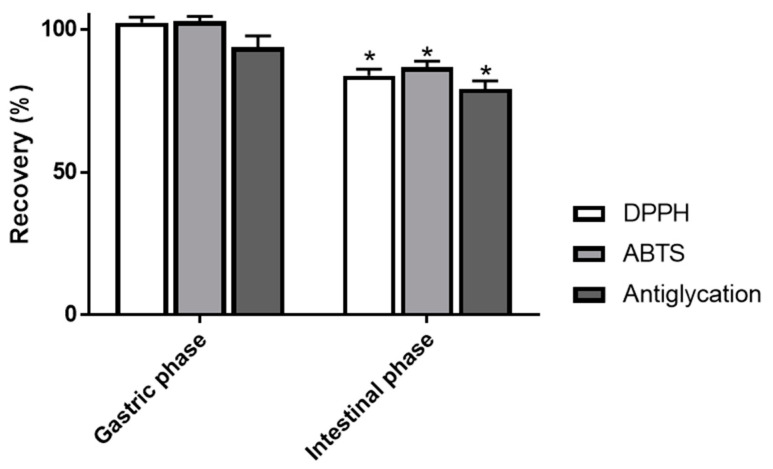
Recovery index of radical scavenging (DPPH, ABTS) and antiglycation activities of *Vaccinium myrtillus* fruit extract after each step of in vitro gastrointestinal digestion. Values are presented as means ± SEM (*n* = 3). * *p* < 0.05 vs. control (undigested matrix). DPPH: 2,2-diphenyl-1-picrylhydrazyl, ABTS: 2,2’-azino-bis 3-ethylbenzothiazoline-6-sulphonic acid.

Consistently with the described stability of anthocyanins in this media, prominent recovery rates of 102.3 ± 1.9 and 102.8 ± 1.7% were respectively recorded for DPPH and ABTS radical scavenging effects (Figure 6). In addition, a subsequent recovery value of 93.7 ± 4.0% was also determined regarding AGEs inhibition activity of AEVM.

On the other hand, intestinal step elicited more substantial variations. Indeed, moderate reductions of around 15% were assessed with the two antioxidant assays. Of note, no significant difference was detected between relative diminutions of DPPH and ABTS scavenging activities (*p* > 0.05). It tends to suggest that hydrogen atom transfer (HAT) and single electron transfer (SET) actions were equally affected by intestinal digestion. Indeed, most authors consider that DPPH assay is mainly associated with a HAT mechanism whereas ABTS reduction is mediated by SET and HAT reactions [34]. Besides, significant reduction of AGEs inhibition activity AEVM was also established, as attested by a recovery index of 79.1 ± 2.8%. Taken together, these results indicate that intestinal step causes a negative effect on antiglycoxidant properties of the studied extract. It should therefore be noted that this impact can be considered as moderate since recovery rates of around 80% have been determined for all activities. Such values might be partly attributed to the unharmed parent anthocyanins that are still occurring after intestinal simulated treatment. However, noteworthy differences have been observed between reductions in chemical contents and antiglycoxidant activities. It strongly suggests that anthocyanin decomposition products are also able to exert such biological activities. Indeed, owing to their above attested phenolic nature, these components are likely capable of scavenging radical entities and exerting antiglycation action. Although it appears that their activities might not be as potent as those of parent anthocyanins, the present results attest that simulated digestion does not profoundly affect antiglycoxidant properties of AEVM.

## 4. Conclusions

In vitro evaluation of the bioactivity of foods and natural products is of major importance to provide a scientific support to their potential physiological effects. Nevertheless, such investigations might not be sufficient to ascertain their health benefits since the bioavailability and the metabolism of their bioactive components are not taken into account. In particular, numerous phenolic derivatives have been reported to be sensitive to gastrointestinal tract conditions, underlining the actual necessity of assessing their digestive fate. By employing a simulated in vitro digestion model, the present study points out the limited bioaccessibility of bilberry anthocyanins and highlights their substantial instability in intestinal conditions. Besides, HPLC analyses revealed noteworthy differences in their digestive fate that mainly depend on the nature of their aglycone moieties. Indeed, cyanidin, peonidin, and malvidin glycosides were shown to be noticeably less altered than petunidin and delphinidin ones. By contrast, it is of major interest to note that the antiglycoxidant properties of the studied enriched extract were well preserved despite the digestive susceptibility of its bioactive compounds. Indeed, prominent recovery rates of around 80% were evaluated for both radical scavenging and antiglycation activities. Taken together, these data indicate that digestive conditions might not deeply impair the potential positive effects of bilberry anthocyanins on oxidative and carbonyl stresses. However, to fully validate the physiological relevance of these antiglycoxidant properties, further investigations will be required to take into consideration intestinal absorption and subsequent metabolization of bioactive components.

## Figures and Tables

**Table 1:** Impact of in vitro gastrointestinal digestion on total phenolic and total anthocyanin contents of *Vaccinium myrtillus* fruit extract.

Assay	Undigested Matrix	Gastric Phase	Intestinal Phase
Total Phenolic Content (mg Gallic acid equivalent/g)	434.8 ± 1.9 ^a^	456.6 ± 7.5 ^b^	438.7 ± 2.1 ^a^
Total Anthocyanin Content (mg Cyanidin 3-*O*-glucoside equivalent/g)	321.8 ± 2.0 ^a^	323.2 ± 5.0 ^a^	127.4 ± 2.4 ^b^

Results are expressed as mean values ± SEM *(n* = 3). Values in the same row sharing identical superscript are not significantly different from each other (*p* > 0.05). Contents were calculated per gram of dry extract.

**Table 2:** Impact of simulated digestion on anthocyanin composition of *Vaccinium myrtillus* fruit extract.

Peak Number	Compound	Gastric Phase (Recovery, %)	Intestinal Phase (Recovery, %)
1	Delphinidin 3-*O*-galactoside	97.6 ± 1.6	18.4 ± 0.4
2	Delphinidin 3-*O*-glucoside	96.7 ± 1.5	16.1 ± 0.4
3	Cyanidin 3-*O*-galactoside	98.0 ± 1.1	40.9 ± 0.7
4	Delphinidin 3-*O*-arabinoside	98.5 ± 2.2	17.4 ± 0.2
5	Cyanidin 3-*O*-glucoside	98.1 ± 1.5	38.2 ± 0.6
6	Petunidin 3-*O*-galactoside	99.2 ± 0.7	30.1 ± 1.4
7	Cyanidin 3-*O*-arabinoside	100.1 ± 2.9	40.0 ± 0.5
8	Petunidin 3-*O*-glucoside	97.7 ± 1.5	30.5 ± 0.5
9	Peonidin 3-*O*-galactoside	98.3 ± 2.9	40.5 ± 0.7
10	Petunidin 3-*O*-arabinoside	98.8 ± 2.6	29.8 ± 0.3
11	Peonidin 3-*O*-glucoside	98.7 ± 1.4	39.3 ± 1.2
11′	Malvidin 3-*O*-galactoside		
12	Malvidin 3-*O*-glucoside	98.3 ± 1.6	39.2 ± 1.1
13	Malvidin 3-*O*-arabinoside	98.4 ± 1.5	41.2 ± 1.4

Recovery ratios were calculated by comparison with undigested matrix values. Results are expressed as mean ± SEM (*n* = 3). 11 and 11’ correspond to co-eluting compounds.

**Table 3:** Impact of in vitro gastrointestinal digestion on radical scavenging and antiglycation activities of *Vaccinium myrtillus* fruit extract.

Assay	Undigested Matrix	Gastric Phase	Intestinal Phase
DPPH scavenging activity (μmol of Trolox eq/g)	2696.5 ± 26.5 ^a^	2758.1 ± 30.0 ^a^	2259.5 ± 70.9 ^b^
ABTS scavenging activity (μmol of Trolox eq/g)	4732.6 ± 54.5 ^a^	4862.5 ± 57.4 ^a^	4102.8 ± 83.5 ^b^
Antiglycation activity (IC_50_, mg/L)	70.41 ± 4.38 ^a^	75.10 ± 3.15 ^a^	89.04 ± 5.24 ^b^

Results are expressed as mean values ± SEM (*n* = 3). Values in the same row sharing identical superscript are not significantly different from each other (*p* > 0.05). DPPH (2,2-diphenyl-1-picrylhydrazyl) and ABTS (2,2’-azino-bis 3-ethylbenzothiazoline-6-sulphonic acid) radical scavenging values are expressed as mg of Trolox equivalent/g of dry extract. Antiglycation activity is expressed as IC_50_ in mg of dry extract/L.
